# The effectiveness of lunchbox interventions on improving the foods and beverages packed and consumed by children at centre-based care or school: a systematic review and meta-analysis

**DOI:** 10.1186/s12966-019-0798-1

**Published:** 2019-04-29

**Authors:** Nicole Nathan, Lisa Janssen, Rachel Sutherland, Rebecca Kate Hodder, Charlotte E. L. Evans, Debbie Booth, Sze Lin Yoong, Kathryn Reilly, Meghan Finch, Luke Wolfenden

**Affiliations:** 1 Hunter New England Population Health, Hunter New England Local Health District, Newcastle, Australia; 2 School of Medicine and Public Health, The University of Newcastle, Newcastle, Australia; 3 Priority Research Centre for Health Behaviour, The University of Newcastle, Newcastle, Australia; 4 Hunter Medical Research Institute, Newcastle, Australia; 5 University Library, Academic Division, University of Newcastle Australia, Newcastle, Australia; 6 Nutritional Epidemiology Group, School of Food Science and Nutrition, University of Leeds, Newcastle, UK; 7 Hunter New England Population Health, Locked Bag No. 10, Wallsend, NSW 2287 Australia

**Keywords:** Diet behaviour, Children, Lunchbox, Systematic review, Packed lunch

## Abstract

**Objective:**

To assess the effectiveness of lunchbox interventions aiming to improve the foods and beverages packed and consumed by children at centre-based care or school; and subsequent impact on children’s adiposity.

**Methods:**

Systematic search of nine databases for controlled trials published in English between 1995-January 2017. Where appropriate, data were pooled in a random effects meta-analysis.

**Results:**

Of the 1601 articles identified, ten studies (centre-based care *n* = 4, school *n* = 6) were included of which eight were RCTs. The impact of interventions on the packing of discretionary foods, sugar-sweetened drinks and other core foods was inconsistent. Meta-analysis of four RCTs trials found a moderate increase in provision of vegetables (SMD = 0.40 95% CI 0.16 to 0.64, *p* = 0.001, I^2^ = 82%; equivalent to a mean difference of 0.28 serves) but not fruit. Four studies reported impact on children’s dietary intake, one reported no significant effect on consumption of discretionary foods, one reported improvements in the consumption of sugar-sweetened drinks and water, and two reported improvements in consumption of vegetables and fruit. Two studies, that were broader obesity prevention interventions, reported no significant impact on adiposity.

**Conclusions:**

There is some evidence that lunchbox interventions are effective in improving the packing of vegetables in children’s lunchboxes, however more robust research is required to determine the impact on children’s dietary intake and adiposity.

**Trial registration:**

PROSPERO 2016: CRD42016035646.

**Electronic supplementary material:**

The online version of this article (10.1186/s12966-019-0798-1) contains supplementary material, which is available to authorized users.

## Introduction

Children who fail to consume sufficient portions of vegetables and fruit, and overconsume energy-dense, nutrient-poor foods and beverages such as confectionary, potato chips and sugar-sweetened drinks (discretionary foods) significantly increase their risk of developing future chronic disease and increase their risk of unhealthy weight gain [1–3]. Evidence suggests that a large proportion of children in high-income countries, including the United States (U.S.), United Kingdom (U.K.), and Australia, consume diets that are less than adequate, with a significant proportion of children’s energy intake coming from discretionary foods [4, 5] and less than 20% of children meeting recommended vegetable and fruit intake [6–9]. As dietary behaviours established in childhood can track through to adulthood, [10] supporting the establishment of healthy dietary habits in childhood has the potential to reduce the burden of obesity and other current and future diet-related disease [11].

Schools and early childhood education and care settings (here after referred to as centre-based care, which include preschools, long day-care services and kindergartens) have been identified as important settings in which to implement population wide interventions to improve child diet and to reduce overweight and obesity, given their continuous and intensive contact with children [12, 13]. Research indicates that children consume between one third [14] to one half [15] of their daily energy intake whilst in schools and centre-based care; providing an opportune time to impact on their dietary intake. Although these settings may provide meals to children, a significant proportion of children in many countries rely on parents and carers to provide all or some of their child’s food and beverages for consumption whilst in attendance. For example, in the U.S. and U.K. it is estimated that on any given day 40–50% of children [16, 17], in both school and centre-based care, bring lunch and/or snacks from home whilst in Australia, Mexico and New Zealand most children bring a packed lunchbox to school [18–20].

Evidence suggests that foods provided by parents for consumption in these settings are not in line with dietary guidelines. For example, Australian observational studies have reported lunchboxes in primary schools have an excess of discretionary foods with the average lunchbox containing 3.1 serves [14]. Similarly, only 1.6% of primary school children surveyed in the U.K. had packed lunches that met nutritional standards, whilst 52–60% of lunchboxes contained sweet and savoury discretionary snacks [21]. Conversely the inclusion of core foods i.e. fruits and vegetables, whole grain cereals, lean meats etc. is notably inadequate with one study identifying that only 14% of the packed lunchboxes of children aged 11–12 years contained a piece of fruit [22]. Studies in centre-based care have found similar patterns. For example, a cross-sectional study conducted in 18 Australian centre-based care services found that; of the 49 children who were observed that brought food from home via a lunchbox, none met the daily dietary recommendations and, children consumed an average of 0.7 serves of discretionary foods [23]. Similarly, a study of 528 pre-school children’s lunchboxes in California found that more than 80% of lunchboxes contained discretionary foods, such as chips and sugar-sweetened drinks, whilst only 16% included vegetables [24]. Globally, these studies demonstrate a need for effective interventions to support the provision of nutritionally balanced lunchboxes.

Whilst studies have suggested that interventions to improve the nutritional contents of lunchboxes are warranted, [14] very little is known regarding the impact of such interventions on improving the contents of children’s lunchboxes. To our knowledge only one systematic review examining the effectiveness of lunchbox interventions has been conducted and included studies published up to the start of 2013 [25]. This narrative review, published in Spanish, included four studies examining the impact of interventions on vegetables and fruit packed in children’s lunchboxes and reported a significant increase in the provision of and consumption of vegetables and fruit within lunchboxes [25]. However, the review did not investigate if such lunchbox interventions impacted on the packing or consumption of other foods (i.e. discretionary foods) and beverages, and on students’ body mass index (BMI) or adiposity. To address these limitations and guide the development and implementation of effective lunchbox interventions, an updated and comprehensive synthesis of current evidence is needed.

## Aims and objectives

The primary aim of the review was to assess the effectiveness of lunchbox interventions aiming to improve the foods and beverages packed and consumed by children attending centre-based care or school. A secondary aim of the review was to assess the effectiveness of these interventions on child adiposity (e.g. weight or BMI) or waist circumference.

## Methods

### Registration

The review was prospectively registered with PROSPERO (CRD42016035646) and is reported in accordance to the Preferred Reporting Items for Systematic Reviews and Meta-Analyses (PRISMA) guidelines [26].

### Eligibility criteria

Studies were eligible for inclusion if (i) participants were children aged 2–18 years. Studies with only children aged less than 2 years were ineligible due to the different nutritional requirements and developmental stages of young children; (ii) interventions included any educational, experiential, health promotion and/or family or structural or policy or legislative interventions that targeted food provided from home for child consumption during attendance at school or centre-based care (either explicitly or as part of a broader obesity prevention intervention); (iii) they included parallel comparison groups e.g. randomised controlled trials (RCTs), controlled clinical trials, non-randomised trials; and (iv) reported outcomes included either a change in the number or proportion of serves, portions, or grams of food provided or consumed as measured by direct observation, surveys or weighed food in lunchboxes. Secondary outcomes for anthropometry could be measured by BMI, BMI percentile, waist measurements or body composition (e.g. per cent body fat, per cent lean body mass or skin folds). There were no restrictions on length of follow-up time or on publication status. Studies were excluded if interventions were targeting the treatment or management of diagnosed diseases or health problems that impacted on child diet or weight, for example, eating disorders, such as anorexia nervosa or bulimia, or overweight or obesity.

### Information sources and search strategy

A comprehensive search strategy was developed in consultation with an academic librarian (DB), and using previously published search filters [27, 28]. Author (DB) conducted databases searches for studies from earliest record until January 312,017 that were available in: MEDLINE, MEDLINE in Process, EMBASE & A + EDUCATION, PsycINFO, CINAHL, Cochrane Central Register of Controlled Trials (CENTRAL), ERIC, Proquest, Scopus. Search strategies were developed in MEDLINE and adapted according to the individual databases (see Additional file 1 for Medline search strategy). To identify additional articles the reference lists of all included studies were screened, lead authors of included studies were contacted, and hand searches of three key behavioural nutrition journals (Public Health Nutrition, International Journal of Behavioral Nutrition and Physical Activity and Health Education Research).

### Study selection

**Fig. 1:**
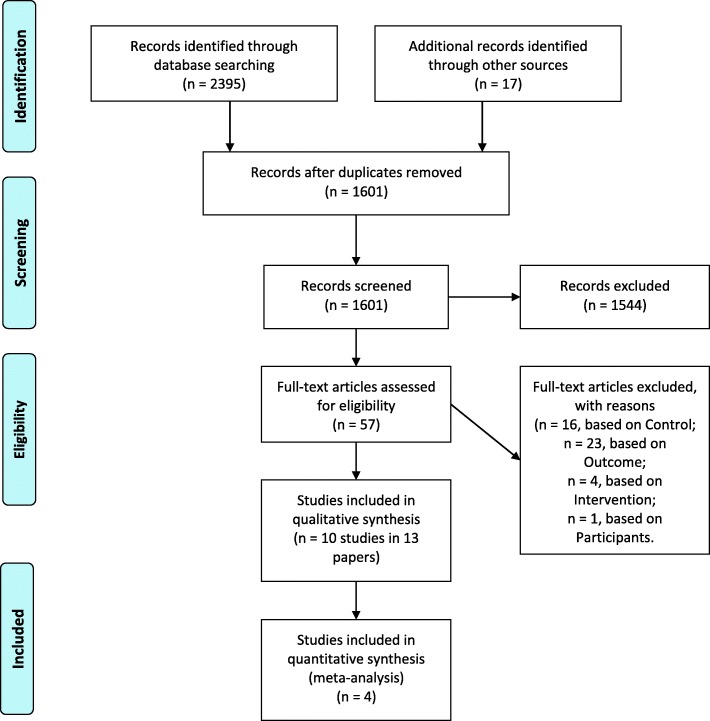
PRISMA diagram

Double independent searching for eligible studies by viewing titles and abstracts was conducted by authors (NN, LJ). The full texts of all potentially relevant studies were obtained and assessed against the inclusion criteria described above by authors in teams of two (NN, RS, MF, LJ). Disagreement regarding the eligibility of a study was resolved by discussion and consensus or consultation with a fifth author (CE). The number of articles at each screening stage is shown in Fig. 1.

### Data collection process

Relevant information was extracted independently from included studies by two reviewers (NN, LJ), using a data extraction tool adapted from Cochrane data collection form for intervention reviews [29]. The following information was extracted: study aim, setting, country, study design, number randomized or allocated to treatment groups (for non-randomized trials), intervention components, duration and theoretical framework, primary outcomes, measures and results (mean and standard deviation (SD) data for all continuous outcomes) and information to assess risk of bias. Any discrepancies during data extraction were resolved through discussion and consensus or consultation with a third reviewer (LW). Where key data were missing from the study reports, we attempted to contact the authors to obtain the information. Any information provided was incorporated into the review as appropriate.

### Risk of bias

Risk of bias (extended criteria for cluster RCTs (C-RCTs)) for the included studies was assessed independently by two reviewers (NN, RH) using the Cochrane risk of bias tool [30]. Each study was assessed as being at ‘high’, ‘low’ or ‘unclear’ risk of bias for: sequence generation, allocation concealment, blinding of participants and personnel, blinding of outcome assessment, incomplete outcome data, selective outcome reporting and ‘other’ potential sources of bias, that is recruitment, loss of clusters, analysis and contamination. Any disagreement was resolved by discussion between the two reviewers.

### Data synthesis

All dietary outcomes were assessed for suitability for pooled analysis with data from trials reporting a comparable outcome measure synthesised in meta-analyses. There were insufficient numbers of studies to pool data from non-randomised trial designs, or dichotomous trial outcomes. If insufficient data were reported to enable inclusion in meta-analysis (i.e. aggregate outcome fruit and vegetable consumption) authors were contacted for relevant information. Available data from the longest follow up period was extracted for synthesis in meta-analyses. We assessed C-RCTs for unit of analysis error. If analyses did not account for clustering of responses within settings, we used intra-class correlation coefficients (ICCs) from similar studies and outcomes to allow calculation of design effects and effective sample sizes to enable pooling in meta-analyses [30].

Where meta-analysis was possible, standardised mean differences (SMDs) were calculated to account for variable outcome measures for each comparison, using the generic inverse variance method, in a random-effect meta-analysis model via RevMan software. SMDs for each comparison were re-expressed as mean differences based on a familiar instrument from a study with the lowest risk of bias in that comparison (e.g. serves of vegetables or fruit provided), by multiplying the baseline standard deviation of the control group by the pooled SMD [30].

Heterogeneity was assessed using the I^2^ statistic. I^2^ values of lower than 50% were deemed to be acceptable levels of heterogeneity. For comparisons where I^2^ values were higher than 50%, pre-specified subgroup analyses were conducted to investigate the source of heterogeneity by setting (centre-based care versus schools). Where pooling was not possible, findings were narratively synthesised according to primary and secondary outcomes.

## Results

### Study selection

Overall 1601 records were screened for eligibility of which 1544 were excluded. Of the remaining 57 papers included in the full-text screen, 44 were excluded as they did not meet our eligibility criteria (see Fig. 1 for PRISMA diagram), leaving 13 papers, reporting on 10 trials included within this review.

### Study characteristics

#### Types of studies

A description of the included studies is shown in Table 1. Of the 10 included trials, three were conducted in the U.S., [31–33] three in U.K., [21, 34–36] two in Australia [37, 38], one in Mexico [39] and one in Israel [40]. Eight of the studies employed cluster randomised-controlled trial designs, [21, 31, 32, 34, 37–40] and two were conducted using quasi-experimental designs [24, 35]

**Table 1:** Summary of included studies

Study name (First author, year published, country) (Reference)	Study Design and Aim	Number Randomised and Sample Size (Age range of sample	Intervention duration: intervention components, theoretical framework.	Follow-up period from baseline data collection	Measures	Primary Outcome/s	Results
SCHOOL INTERVENTIONS							
Nutritional Intervention to Improve the Quality of Lunchboxes Among Mexican School Children (Diaz-Ramirez, 2016, Mexico)	Cluster randomised controlled trial Evaluate the effects of an intervention program to improve the quality of the foods in the lunchboxes.	4 schools (2 IG, 2 CG) 973 students (8–14 years)	8 weeks: written information to parents, classroom posters for children Standalone intervention No theoretical framework identified. CONTROL: Comparison condition not described	6 months	Lunchbox registry for three non-consecutive days, using a pre-validated comparison list.	Proportion of lunchboxes with ≤276 kcal, fruits and/or vegetables, no unhealthy prepared foods, no unhealthy solids and liquids, meeting the established guidelines of an adequate lunchbox and a healthy lunchbox. Provision only.	Total fat and sugar were lower in int (*p* = 0.003 and 0.002); Increase in the number of students who met the criteria of an adequate lunchbox (19% IG vs 10% CG (*p* = 0.002).
SMART lunch box (Evans, 2010, UK)	Cluster randomised controlled trial (RCT) Conduct the first known cluster randomised controlled trial to improve the contents of packed lunches using an intervention named the “SMART” lunch box, thereby bringing packed lunches more in line with school meals using current guidelines.	89 schools (44 IG, 44 CG)^a^ 1294 students (8–9 years)	5 months: lunch bag and two food boxes together with supporting materials for parents and children. Standalone intervention No theoretical framework identified. CONTROL: Usual practice.	12 months	Weighed food record using specifically designed assessment tool. Assessed before and after lunch.	Weights of food groups provided (sandwiches, fruit, vegetables, dairy foods, savoury snacks and confectionery). Levels of 14 nutrients provided. Provision and consumption^b^	Higher weights of food types in IG (fruits, vegetables, dairy foods and starchy foods other than bread in lunchboxes. Weights of savoury snacks was lower in IG. Higher levels of Vit A and folate in IG.
Great Taste, Less Waste (GTLW) (Goldberg, 2015, US)	Cluster randomised controlled trial (RCT) – 3 arm trial GTLW (a communication campaign, capitalized on the synergy between healthy eating and eco-friendly behaviours) would improve the quality of foods from home more than a nutrition-only campaign (F2C) or a control.	15 schools (3 IG-GTLW, 3 IG-F2C, 3 CG) 582 students (8–10 years)	22 weeks: curriculum lessons and workbook, campaign kit, monthly parent newsletters, poster contest, monthly email to teachers. With a nutrition-eco approach Standalone intervention. The Theory of Reasoned Action (TRA) and Social Cognitive Theory IG-F2C: same as GTLW, with a nutrition only focus. CONTROL: Waitlist control.	7 months	Digital photography of lunch and snacks supplemented by a checklist.	Mean servings of fruits, vegetables and SSDs; mean prevalence of packaged items; prevalence of food items of interest. Provision only	No significant differences were observed between groups in mean servings of fruit, vegetables or SSDs or mean prevalence of packaged items.
Food Dudes Ireland (Horne, 2009, UK)	Cluster randomised controlled trial Evaluate the effectiveness of the Food Dudes intervention in the context of the Irish school system.	Two schools (1 IG, 1 CG) 435 students (4–11 years)	12 months (16 day intervention and 12 months maintenance): videos featuring Food Dudes, small rewards for eating fruits and vegetables, Food dudes homepack Standalone intervention. No theoretical framework identified. CONTROL: No intervention.	12 months	Visual estimation of fruit and vegetable portions in lunchbox. Assessed before and after lunch.	Parental provision and consumption of fruits and vegetables (in grams) in the lunchbox. Provision and consumption	Parents from intervention schools provided and their children consumed more fruit, vegetables and juice relative to baseline and control. Provision of FJV 103 g vs 71 g; consumption of FJV 71 g vs 47 g.
Food Dudes England (Upton, 2014, UK) (Upton, 2012, UK)	Quasi-experimental Investigate the effectiveness of the Food Dudes Program in increasing primary-school children’s fruit and vegetable consumption for both home- and school-supplied meals; and to establish the extent to which the programme is able to influence the long-term maintenance of any behaviour changes which were observed.	15 schools (8 IG, 7 CG) 2443 students (4–11 years)	16 day intervention plus 12 month maintenance phase: series of DVD episodes of the Food Dudes’ adventures, letters to students from Food Dudes, rewards for tasting fruit and vegetables and then for consumption, Food Dudes home pack. (Maintenance phase – consumption is encouraged but with less intensity than intervention phase). Standalone intervention. No theoretical framework identified. CONTROL: No intervention.	12 months	Visual estimation from digital photography of lunchbox using previously validated guidelines. Assessed before and after lunch.	Average daily consumption of fruits, vegetables and high fat/sugar foods (in portions) for children who consumed home-supplied lunches. Consumption	No significant difference in in the consumption of fruits and vegetables and high fat/sugar foods from home supplied lunches in the intervention group.
School based intervention on Nutritional Knowledge and Habits of LSES Children in Israel (Kaufman-Shriqui, 2016, Israel)	Cluster-randomized trial Examine the effects of a school-based comprehensive intervention on nutrition knowledge, eating habits, and behaviours among low socioeconomic status (LSES) school-aged children.	11 schools (7 IG, 4 CG) 238 children (4–7 years)	3 months: 10 weekly nutrition lessons for children; weekly newsletters for parents; 3 meetings for parents; training for teachers. Broader nutrition and PA intervention. Ecological Model. CONTROL: PA only.	6 months	Observation to calculate a lunchbox score based on five categories (a score of 0 or 1 for each category and then added together to calculate a total score out of 5).	Quality score of packed lunch from 0 to 5. Provision	Significant change in the overall packed lunch score over time between the IG and CG (1.16 vs 0.41; *p* < 0.0001)
CENTRE-BASED CARE INTERVENTIONS							
Munch and Move (Hardy, 2008, Australia)	Cluster randomized controlled trial Evaluate “Munch and Move”, a low-intensity, state-wide, professional development program designed to support early childhood professionals to promote healthy eating and physical activity among children in their care.	29 preschools (15 IG, 14 CG) 430 children (3–5 years)^c^	20 weeks: professional development for staff, resource provision (manual, fact sheets, games and small grant), project officer support visits. Broader nutrition and PA intervention. No theoretical framework identified. CONTROL: Waitlist control (During intervention, received health information on non-related topics).	20 weeks	Weighed food record of all food and beverages provided.	Mean serves of fruits, vegetables, snacks and sweetened drinks; Lunchboxes with > 1 serve of extra food / drinks; Proportion of lunchboxes meeting categories of balanced, overloaded with extras, unbalanced. Provision	Significant reduction in sweetened drinks by 0.13 serves (*p* = 0.05).
Lunch is in the Bag (Roberts-Gray, 2016, United States)	Cluster randomised controlled trial Assess the efficacy of the “Lunch is in the Bag” intervention to increase parents’ packing of healthy bag lunches for young children.	30 Early Care and Education Centres (15 IG, 15CG) 633 parent-child dyads (3–5 years)	5 week (plus 1 week booster): parent handouts/newsletters, teacher/child classroom activities, parent/child activity stations, calendar, workshop and materials. Standalone intervention. Social Cognitive Theory, Theory of Reasoned Action and Ecological Approach. CONTROL: Waitlist control.	28 weeks	Direct observation of contents of lunchboxes using a structured food record.	Servings of fruit, vegetables, whole grains, refined grains, protein foods and dairy in the children’s parent-packed bag lunches. Provision	Increased the number of servings of fruit and of whole grains.
Lunch is in the Bag (Sweitzer, 2010, United States) (Briley, 2012, United States) (Sweitzer, 2014, United States)	Quasi-experimental controlled trial Pilot test of a centre-based program to encourage parents of preschool aged children to pack one serving of fruits, vegetables, and wholegrains every day in sack lunches sent from home.	Six childcare centres (3 IG, 3CG) 132 parent-child dyads (3–5 years)	5 weeks: parent handouts, classroom activities, educational stations, teacher training. Standalone intervention. Intervention Mapping, Theory of Planned Behaviour, Social Cognitive Theory. CONTROL: Comparison condition not described.	6 weeks	Direct observation of packed food items using a Food Observation Record.	Number of servings of fruits, vegetables, and whole grains in lunches children brought from home. Provision	Increased the number of servings of vegetables and whole grains
Tooty Fruity Vegie (Zask, 2012, Australia)	Cluster randomised controlled trial Decrease overweight and obesity prevalence among children by improving fundamental movement skills, increasing fruit and vegetable intake and decreasing unhealthy food intake.	31 preschools (18 IG, 13 CG) 560 children (29–73 months)	10 months: Food and nutrition policy, parent workshops and resources, consistent messages for children through puppets, stories, role plays, cooking, staff as role models and positive reinforcement, increase accessibility to water. Broader nutrition and PA intervention. CONTROL: Waitlist control	21 months	Visual estimation of fruits, vegetables and EDNP foods and drinks.	Number of fruit and vegetable serves, proportion of children with 0, 1, 2+ EDNP food items in lunchbox. Provision	Significant increase in the mean number of Fruits and vegies.

^a^One school withdrew before randomisation. ^b^Paper only reports on provision but consumption data obtained from author. ^c^259 students in lunchbox analysis. *IG* Intervention Group, *CG* Control Group, *SSD* sugar-sweetened drinks, *EDNP* energy dense nutrient poor

.

#### Participants

Four trials were conducted in centre-based care [24, 32, 37, 38] with the number of participating centres ranging from six to 31. These trials recruited samples of between 132 and 560 children with the age of participants ranging from 3 to 6 years. Of the six trials conducted in schools, [21, 31, 34, 35, 39, 40] the number of participating schools ranged from two to 89. These trials recruited samples of between 238 and 2443 students with the age of participants ranging from 4 to 14 years.

#### Interventions

All 10 of the included trials utilised multicomponent interventions (employed two or more intervention strategies). All trials included intervention strategies to increase parent knowledge via delivery modes such as pamphlets, newsletters, posters or parent workshops. The majority of interventions (8/10) included an education component for children through videos, games, curriculum or activities [24, 31, 32, 34, 35, 37, 38, 40]. Four interventions, all school based, provided physical resources (e.g. lunch packs, containers) to support the packing of healthy lunchboxes [21, 31, 34, 35] while two provided incentives for children to taste vegetables and fruit [34–36]. Only two interventions, both centre-based care studies, incorporated the development of a policy and the communication of this to parents [37, 38]. Seven of the studies were stand-alone lunchbox interventions [21, 24, 31, 32, 34, 35, 39] whilst three were part of a larger child obesity prevention program, two occurring in centre-based care which included strategies to support centres to deliver physical activity (e.g. manuals, training, equipment) or information to parents targeting small screen recreation reduction [37, 38] and one in schools which included 15 × 45 min physical activity delivered to children by trained professionals [40]. Intervention duration across the studies ranged from five [24] to 8 weeks [39], 4 to 7 months, [31, 32, 37–40] and up to 12 months [34, 35] (see Table 1).

#### Outcomes

Follow-up data collection ranged from; 6 weeks post-baseline in one trial, [24] five to 7 months in five trials, [31, 32, 37, 39, 40] 12 months in three trials, [21, 34, 35] and 21 months in the remaining trial [38]. Of the eight studies that examined the impact of interventions on the nutritional content of foods and beverages provided from home [21, 24, 32, 34, 37] two studies reported on vegetables and fruit as a combined result, [35, 38] three reported on discretionary foods including snacks, or confectionary; [21, 37, 38] three reported on sugar-sweetened drinks; [21, 31, 37]; and three reported on the proportion of lunchboxes meeting a pre-defined category that is “adequate/ healthy” [39],“balanced/ overloaded with extras/ unbalanced” [37] or a quality score for the packed lunch [40]. One study also reported on the mean weights of sandwiches, dairy foods, and other starchy foods provided [21].

Only three studies reported on the impact of the intervention on child dietary intake of packed foods [21, 34, 36]. Two reported on the mean weight in grams of vegetables and fruit individually consumed [21, 34, 36] and one study [36] reported the combined serves and grams of vegetables and fruit consumed, and serves of high fat and sugar foods.

Lunchbox contents were measured via visual estimation [24, 32, 34, 38, 40] or digital photography [31, 36] which some studies supplemented with a food observation checklist [24, 31, 32] or a weighed measure [21, 37]. In one study [39] it was unclear what method was used. Children’s dietary intake was measured by observing lunchboxes before and after school food breaks to see what was consumed [21, 34, 35]. Two studies, one an obesity prevention intervention in centre-based care [38] and one a nutrition and physical activity intervention in schools [40] included an objective measure of adiposity (BMI Z-score and mean waist circumference).

#### Comparisons

Two trials compared intervention strategies against no intervention, [34, 35] one against usual practice [21], one against a physical activity intervention only [40] and three against wait-list control, [32, 37, 38] of which one trial provided the control group general health information on an unrelated topic while on wait-list [37]. One trial included three trial arms, [31] i.e. two interventions (Great Taste Less Waste (GTLW) and Foods 2 Choose (F2C)) and one control, of which both intervention conditions sought to increase children’s packing of vegetables and fruit. Both interventions were virtually identical however GTLW included “eco” messages that linked healthy eating to the environment. Two trials did not describe the comparison condition [24, 39].

### Risk of bias

**Fig. 2:**
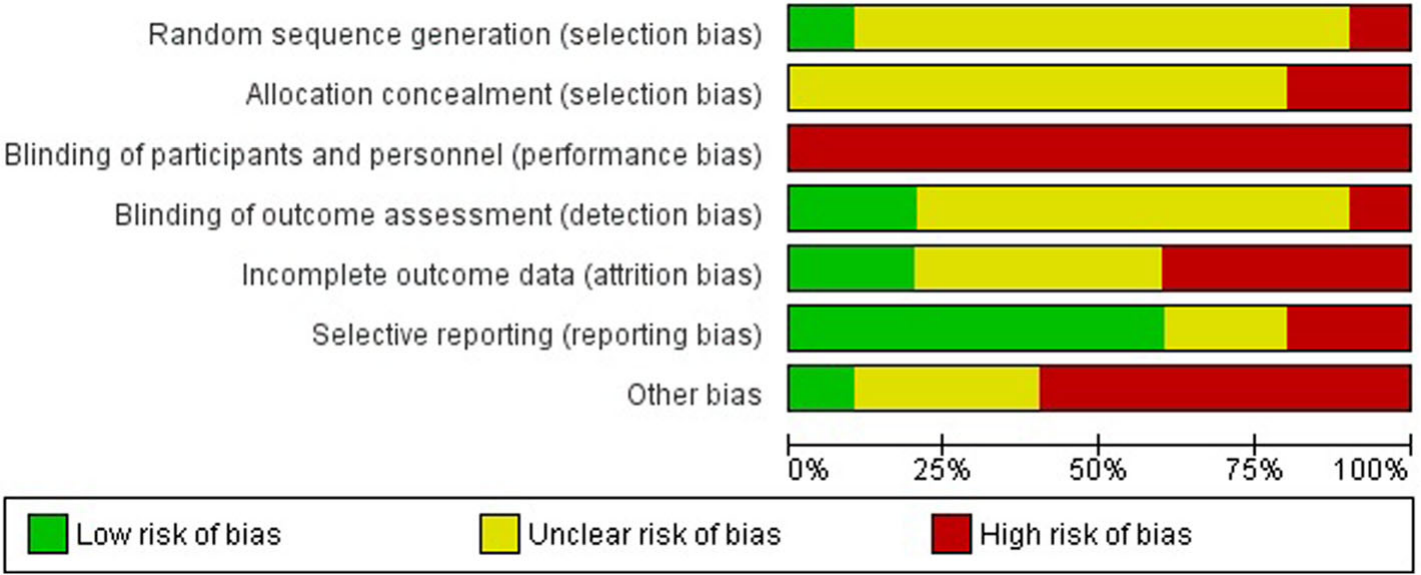
Risk of bias graph

**Fig. 3:**
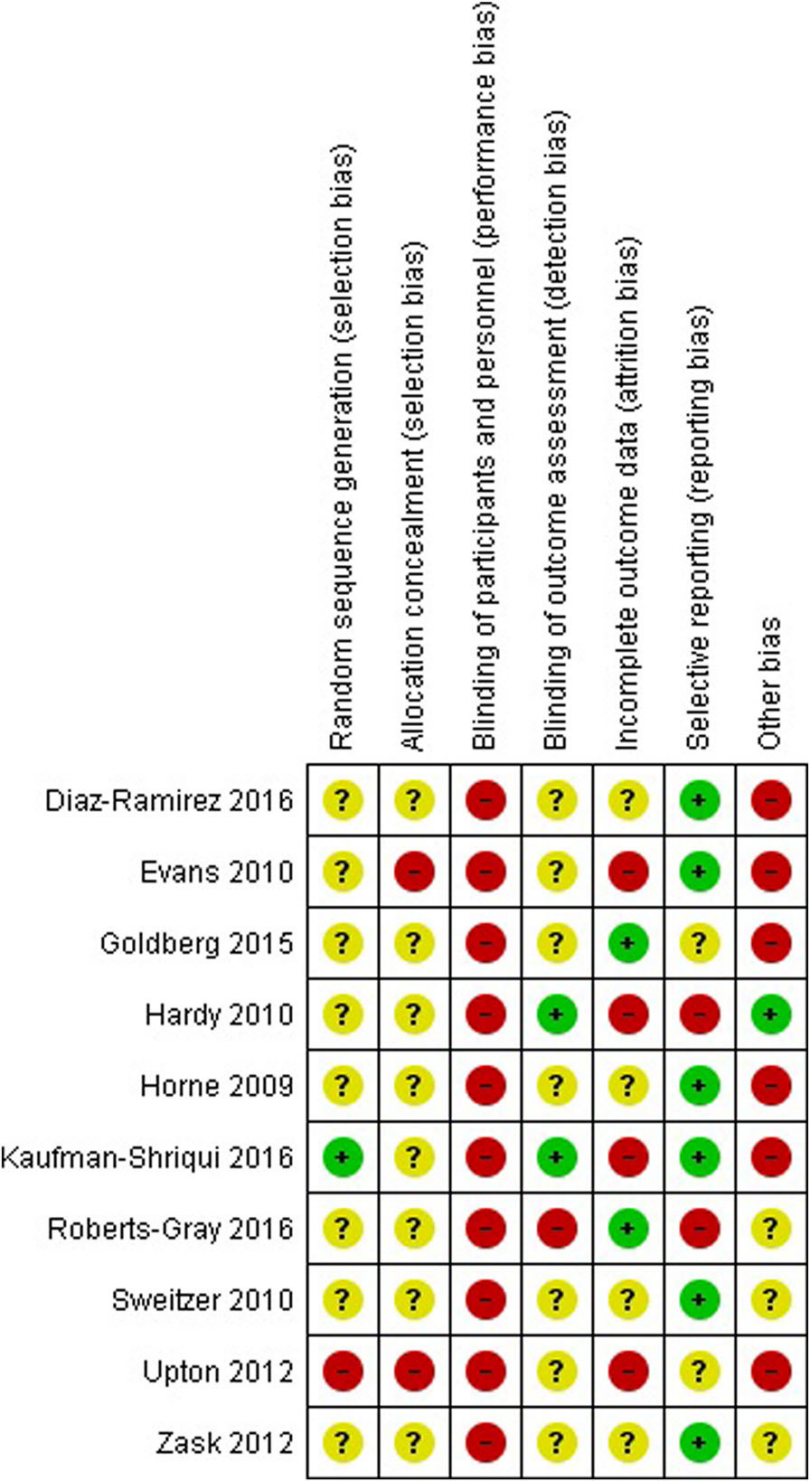
Risk of bias summary

Figures 2 and 3 summarize the risk of bias for each of the studies. Information related to random sequence generation was assessed as low in one study, [40] unclear in eight of the nine included studies [21, 24, 31, 32, 34, 37–39], and high risk for the other study as schools were purposefully selected to receive the intervention or act as control [35]. As allocation concealment was not possible in two studies they were assessed at high risk of bias [21, 35] whereas the other eight were unclear. All of the studies were rated as being at high risk for performance bias given participants or personnel delivering the intervention were not blinded to intervention groups. Two studies reported that outcome assessors were blinded to group allocation and therefore were assessed at low risk of bias [37, 40], one study was high risk as data collectors undertaking outcome assessments were not blinded to group allocation [32], and unclear risk for the remaining seven studies. Four studies [21, 35, 37, 40] were rated as having high risk of bias due to incomplete outcome data due to large attrition (> 20%), two as low [31, 32] and unclear in the remaining four studies [24, 34, 38, 39]. Selective outcome reporting was rated as high risk of bias in two trials where not all primary (e.g. food groups and quantities of foods) [37] or secondary outcomes (e.g. BMI) [32] reported in the published study protocol or trial registration were included in the publication, low risk of bias in one study [40] and unclear risk of bias in the remaining seven trials. Seven studies were assessed as having high risk of other biases (related to not adequately describing if clustering was accounted for in analysis, [24, 34, 39] randomisation after baseline data collection, [31] or loss of clusters following randomisation. [21, 35, 40]), one study was rated at low risk of bias [37] and the remaining two were unclear [24, 38].

### Effects of interventions

#### Impact of interventions on the nutritional content of foods and beverages provided from home (i.e. what is packed)

**Fig. 4:**
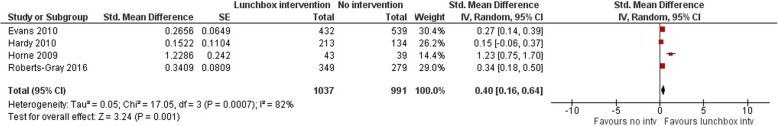
Forest plot – provision of vegetables

**Fig. 5:**
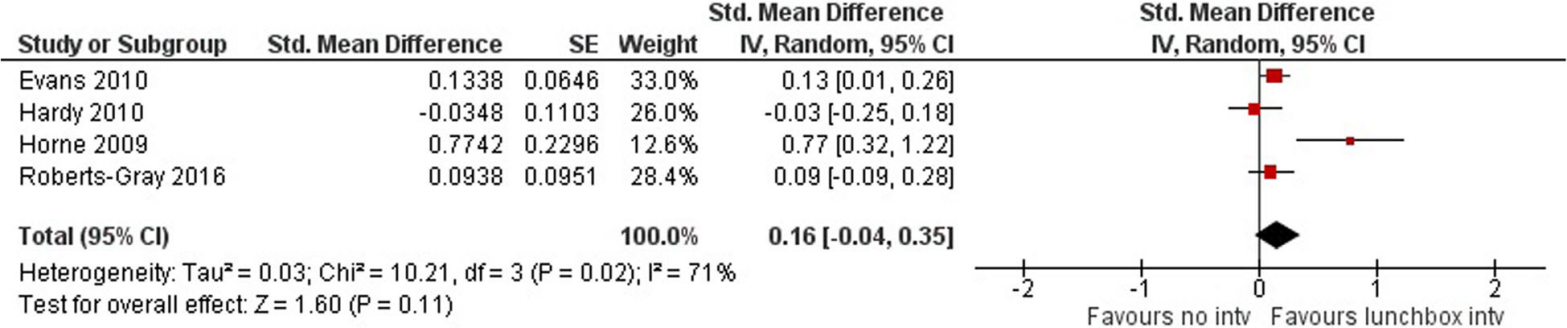
Forest plot – provision of fruit

*Fruit and vegetables:* Meta-analyses of 2792 participants, from four studies (two in centre-base care [32, 37] and two in schools [21, 34]), revealed an overall significant increase in the provision of vegetables (SMD = 0.40 95% confidence interval (CI) 0.16 to 0.64, *p* = 0.001, I^2^ = 82%; equivalent to a mean difference of 0.28 serves) (Fig. 4) but not fruit (SMD = 0.16 95% CI -0.04 to 0.35, *p* = 0.11, I^2^ = 71%) (Fig. 5). Subgroup analyses were conducted on the basis of setting to investigate the source of heterogeneity. For the provision of vegetables, a overall significant increase remained in centre-based care (SMD = 0.26 95% CI 0.08 to 0.44, *p* = 0.005, I^2^ = 47%; equivalent to a mean difference of 0.18 serves) but not schools (SMD = 0.72 95% CI -0.22 to 1.66, *p* = 0.13). For the provision of fruit, there was no overall effect in centre-based care (SMD = 0.04 95% CI -0.10 to 0.18, *p* = 0.59) or schools (SMD = 0.42 95% CI -0.21 to 1.04, *p* = 0.19).

The other four non-randomised trials that measured the packing of vegetables or fruit were not included in the meta-analysis. Two quasi-experimental trials, one conducted in Australian pre-schools [38] and one in U.S. pre-schools, [24] reported significant effects of the intervention on the provision of vegetables and fruit. The Australian study reported an improvement in the number of vegetables and fruits provided (combined) (mean difference = 0.61 serves, standard error (SE) = 0.14, *p* = 0.001) whilst the U.S. study reported a significant increase in mean number of servings of vegetables 0.344 (SE = 0.100, p 0.001) but not fruit (mean serving 0.065, SE = 0.124, p 0.600). Similarly, a trial in two schools in Ireland, reported that children in the experimental school were provided with significantly more fruit, vegetable and juice in their lunchboxes than control children (t = 3.5, *p* < 0.001) [34]. Another trial undertaken in Mexican schools reported a no significant effect on the vegetable and fruit content of lunchboxes of intervention vs control students (39.0% vs 37.0% *p* = 0.57) [39].

*Discretionary foods including snacks, confectionary* - Four studies reported on the impact on the provision of discretionary foods [21, 32, 37, 38]. Of those, one C-RCT, conducted in centre-based care as part of a broader child obesity prevention program, reported significant effects of the intervention on the proportion of children that had lunchboxes with no energy-dense nutrient-poor food items (difference at follow-up 29.1%; SE not able to be calculated, *p* < 0.001) [38]. Conversely, the other study in centre based care, also delivered as part of a broader child obesity prevention program, found no significant impact on lunchboxes containing serves of snacks (mean difference = 0.06 (95% CI -0.34 to 0.46) *p* = 0.75); or one or more serves of extra foods (odds ratio = 0.90 (95% CI 0.48 to 1.70) *p* = 0.74) [37]. One school-based intervention reported mixed results, with a significant weight reduction in the provision of savoury snacks i.e. grams of crisps and other salted snacks (mean difference − 2.8 95% CI -5.5 to − 0.2 *p* = 0.04) but no reported change in the weight of confectionary (foods containing chocolates, cereal bars and sweets) provided (mean difference-2.1 95% CI -5.6 to 1.5 *p* = 0.26 [21]. The other school based study, also reported mixed results, with no impact on the provision of chips but found a significant impact on the servings of sweets (fruit drinks, cookies and candy) (mean difference − 0.43 servings, SE = 0.11, *p* < 0.001) [32].

*Sugar-sweetened drinks*- One study in centre-based care reported significant reductions in the provision of sugar-sweetened drinks by 0.13 serves (approximately 46mls; 95% CI -0.27 to 0.002; (*p* = 0.05)) [37] while two studies in schools reported no significant change in weight of sugar-sweetened drinks provided (mean difference = − 5.0 (95% CI -34.3 to 24.4 p = 0.74) [21] or mean servings of sugar-sweetened drinks provided [31] (intervention vs control *p* = 0.98; intervention vs nutrition only *p* = 0.80).

*“Healthy” lunchboxes*- Three studies used predefined categories to measure the impact of interventions on the contents of children’s lunchboxes [37, 39, 40]. One study in centre-based care [37] reported no significant change in the provision of “balanced” lunchboxes (that is containing at least a sandwich or home cooked meal and either fruit or vegetables, with the allowance of one extra serve of food or beverage) (difference at follow-up = 0.85 (95% CI 0.35 to 2.25) *p* = 0.72). One study in schools [39] reported a significant difference in the proportion of intervention students that had an adequate lunchbox vs. controls (19.1% vs 9.6% *p* = 0.002) (that is containing < 276 cal, vegetables and/or fruits and an item prepared at home) and, although the relative effect size was large there was no significant difference for those that had a healthy lunchbox (that is containing vegetables and/or fruits, water and no unhealthy foods) (10.2% vs 5.9% *p* = 0.09). Whilst the remaining study, also in schools, reported a significant improvement in the packed lunch score at follow-up between intervention and control (1.16 ± 0.16, 120% vs 0.41 ± 0.18, 42% *p* = < 0.001) [40].

*Other foods (including sandwiches, dairy foods, whole grains)*- Three studies, one school-based study [21] and two centre-based care studies [24, 32] examined the impact on the provision of other core foods. The school-based study reported a significant difference in the mean weight of dairy food i.e. cheese and milk based desserts (mean difference 8.4 g 95% CI 2.0 g to 14.9 g *p* = 0.01) and starchy foods other than bread (mean difference 7.4 g 95% CI 0.5 g to 14.g4 *p* = 0.04) provided to intervention children compared to control. However no difference was found in the mean weight of sandwiches (mean difference 3.2 g; 95% CI -4.7gto 11.1 g; *p* = 0.43) and unsweetened drinks (fruit juice and milk) provided (mean difference = 13.6 g; 95% CI -17.2 g to 44.3 g; *p* = 0.39) [21]. The two studies in centre-based care, examined the impact on the provision of wholegrains, with both studies showing an increase in the mean number of servings of wholegrains packed compared to control (0.34 servings; SE = 0.13; *p* = 0.009) [24, 41] and (0.49 servings; SE = 0.15; *p* = 0.001) [32]. In addition, the study by Roberts-Gray, [32] found no impact on the provision of protein-based foods (e.g. meats, nuts, etc.) but showed an increase in the servings of dairy which the authors attributed to a decrease in provision in control centres, not an increase in intervention centres (0.14 servings; SE = 0.05; *p* = 0.011).

#### Impact of interventions on child dietary intake

Three school-based studies reported on the impact of interventions on children’s dietary intake of vegetables and fruit [21, 34–36] one being the pilot [34] for a larger trial [35]. The pilot study by Horne et al. [34] reported a significantly higher mean consumption of fruit, vegetable and juice among intervention students compared to control (t = 3.7, *p* > 0.001) [34]. Conversely, the larger trial by Upton et al., [35] found no change in fruit and vegetables consumed by intervention students at 3-months compared to baseline, however a non-significant decrease at 12 months compared to baseline (d = − 0.16, 95%CI -0.30,0.01). In the control schools however child fruit and vegetable consumption was significantly higher at 3 months compared to baseline (d = 0.26, 95%CI -0.12,0.38) but not at 12 months (d = 0.05, 95% CI -0.08,0.16). The study by Evans et al. [21] found a significant improvement in the consumption of vegetables (6.2 g, SE = 1.8 g, 95% CI 2.7–9.8 g) but not fruit (9.0 g, SE = 5.7 g, 95% CI -2.5 to 20.4 g). The school based study by Upton et al., [36] also reported on the impact of the intervention on child consumption of high fat (> 17.5 g of total fat per 100 g) and high sugar (> 22.5 g of total sugars per 100 g) foods and found no intervention effect between groups.

#### Secondary outcome i: impact of interventions on child adiposity (weight or BMI)

One study, [38] an obesity prevention intervention in centre-based care that included a range of strategies in addition to those targeting the content of student lunchboxes, found a significant adjusted difference on BMI z-score (− 0.15, SE =0.07, *p* = 0.022*)* and waist circumference (− 0.80 cm, SE = 0.35, *p* = 0.020) among children attending intervention services relative to control. However the intervention had no overall significant effect on overweight and obesity prevalence (12.5% among control vs 11.5% amount intervention at follow-up). Similarly, a nutrition and physical activity intervention in schools [40] reported a reduction in 0.1 points in BMI z-score in both intervention and control arms, which they attributed to both intervention and control receiving the physical activity intervention.

## Discussion

This review is the first to report on the effectiveness of lunchbox interventions aiming to improve the foods and beverages packed and consumed by children attending centre-based care or school and subsequent impact on adiposity. Data pooled from four of the 10 included trials suggest that to date interventions have had a modest impact on the provision of vegetables in children’s lunchboxes. Evidence for the effectiveness of interventions on what is packed in children’s lunchboxes in relation to discretionary foods, sugar-sweetened drinks or other core foods were equivocal. Similarly, of the few trials reported the effects of interventions on child dietary intake and adiposity findings were mixed. Such findings suggests that interventions targeting the foods packed for child consumption at school or childcare services can have positive effects, however future research is warranted.

Interestingly, meta-analysis results identified improvements in the contents of children’s lunchboxes related to the provision of vegetables, but not fruit. There was also evidence that improved provision of vegetables packed for children also led to increased vegetable consumption. Given the small proportion of children that meet recommended daily serves of vegetables these findings are encouraging as studies report child dietary behaviours are determined, in part, by the availability and exposure to heathy foods [7, 42]. The findings, however, are in contrast to the findings of reviews of school based vegetable and fruit programmes that typically report greater effects on fruit rather than vegetable intake [43]. Previous programmes to improve vegetable and fruit intake tend to focus on fruit provided as a snack. Potentially, interventions targeting child lunchboxes may provide greater opportunity to increase vegetable intake through modifying snack and meal (lunch) eating occasions. While the effects of vegetable provision were reported, fruit was more frequently packed and consumed by children in the included trials thus potentially explaining the null effect. It does therefore suggest more comprehensive approaches to improve vegetable intake are warranted, such as enhancing the school environment to promote children’s liking and expectation of vegetable consumption at lunchtime.

The findings for the provision of discretionary foods, sugar-sweetened drinks and other core foods were less consistent. These results suggest that removing less healthy items from children’s lunchboxes, such as sugary drinks and sweet and savoury snacks, may be more challenging. As parents are primarily responsible for the packing of lunchboxes of children, greater formative evaluation with parents regarding the barriers to removing such foods may be required to improve the development and effectiveness of future interventions targeting discretionary foods. This may include addressing parents’ concerns regarding time, cost or food safety [44]. Additionally, improvements to the availability of healthy foods in other environments including supermarkets, quick service restaurants and sporting clubs as well as to the promotion and marketing of food to children may be required to improve child intake of these foods. For example, progress has been made in the UK on reducing portion sizes of sweet and savoury snacks as part of the childhood obesity strategy [45].

This review identified a number of opportunities to strengthen the effects of existing interventions, particularly in relation to the packing of discretionary foods. While all included studies in the review incorporated a parent component, many simply relied on passive information dissemination strategies which are typically reported to have limited reach and are inadequate to change behaviour [46]. The use of active intervention strategies with the capacity to deliver a variety of evidence based behavioural change strategies (e.g. electronic based interventions) may be more likely to enhance intervention effects. Furthermore, setting-based interventions that undertake a comprehensive approach to improving child health as encouraged by such frameworks as the health promoting schools framework may improve the impact of interventions. For example, trials examining lunchbox interventions supported by explicit school policies and related teaching curriculum appear warranted.

There are a number of strengths to this review. A comprehensive search strategy, utilising robust review methods was undertaken and where possible the estimates of intervention effects were quantified by using meta-analysis adjusting for clustering of children within centre-based care and schools where necessary. However, a number of limitations are worth considering when interpreting the review findings. Only studies published in English were included, which may have excluded other efficacious studies. A number of the characteristics of included studies were consistently assessed as at high risk of bias. Of concern was the lack of random sequence generation and blinding of outcome assessors, which may reduce the confidence of the individual studies trial findings. The longest follow-up period within each study was used in the meta-analysis, however, it is possible that intervention effects may have attenuated in studies with longer follow-up periods than those with shorter follow-up periods. Furthermore, as most of the trials had follow-up periods of less than 12-months it is not possible to know if intervention effects were sustained over longer periods of time. High levels of heterogeneity were also evident from forest plots. The source of heterogeneity was unclear and could be due to differences in population, intervention, outcome or other methodological factors including those not reported in the included trials. The small number of eligible trials precluded examination of heterogeneity by these factors. The meta-analysis is limited by having only a small number of studies, all of which were multi-component interventions, with various outcomes of interest. Whilst subgroup analysis is included, the heterogeneity of these studies should be considered when interpreting these findings. The external validity of the review findings may also be limited given different school food environments internationally. For example, it is also possible that in countries where approximately half of children have a school meal and half take in a packed lunch (such as the UK) specific types of intervention programmes would have a different impact compared to the same intervention introduced in a country where generally no school meal is provided (such as Australia). More trials are needed to confirm these findings. Despite these limitations, given the foods that children bring from home to eat at centre-based care or school, contribute significantly to their daily energy intake, interventions targeting children’s lunchboxes remain promising as a strategy to improve child public health nutrition.

## Conclusion

Interventions to improve the diet quality of packed lunches in children have a modest impact in improving provision of vegetables. However, improvements in other foods such as sugary drinks and other sweet and savoury snacks are not consistent between studies. Given the significant influence parents and caregivers have on the contents of children’s lunchboxes, interventions should continue to engage parents through active intervention strategies and report the reach of such strategies. Future policies related to children’s packed lunches should aim to further increase the success of improving packed lunches through improvements in the food environment such as increasing the availability of healthy foods and reductions in the marketing of sweet foods and drinks and savoury snacks. The potential concern of any public health intervention is widening health disparities. Future lunchbox interventions should aim to report on intervention effects by socio-economic status of children to ensure this is avoided. Given the limited number of studies that assessed child adiposity future more robust trials are required to investigate the potential impact of lunchbox interventions on child diet, weight and BMI.

## Additional file

Additional file 1: Medline search strategy. (DOCX 15 kb)

## Abbreviations

BMI: Body Mass Index
GRADE: Grading of Recommendations, Assessment, Development and Evaluations
PRISMA: Preferred Reporting Items for Systematic Reviews and Meta-Analyses
RCT: Randomised Controlled Trials
U.K.: United Kingdom
U.S.: United States
