# Supplementary material for: The effectiveness of lunchbox interventions on improving the foods and beverages packed and consumed by children at centre-based care or school: a systematic review and meta-analysis
Source: Int J Behav Nutr Phys Act. 2019 Apr 29;16:38. doi: 10.1186/s12966-019-0798-1 (PMC6489330; doi:10.1186/s12966-019-0798-1)
Supplement: Supplementary file 1 — Medline search strategy. (DOCX 15 kb) [file 12966_2019_798_MOESM1_ESM.docx]

**Database(s): MEDLINE 1946 to Present with Daily Update 
Search Strategy:**

| **#** | **Searches** | **Results** |
| --- | --- | --- |
| 1 | Schools/ | 27853 |
| 2 | Schools, Nursery/ | 1377 |
| 3 | ((primary or elementary or middle or junior or high or secondary) adj (school* or student*)).mp. | 42209 |
| 4 | kinder*.mp. | 19382 |
| 5 | Child, Preschool/ | 786869 |
| 6 | (pre-school* or preschool*).mp. | 789600 |
| 7 | Child Day Care Centers/ | 4476 |
| 8 | (childcare* or child care*).mp. | 10254 |
| 9 | (daycare* or day care*).mp. | 12709 |
| 10 | early child*.mp. | 16844 |
| 11 | (nursery or nurseries).mp. | 9803 |
| 12 | 1 or 2 or 3 or 4 or 5 or 6 or 7 or 8 or 9 or 10 or 11 | 882171 |
| 13 | (lunchbox* or lunch box*).mp. | 46 |
| 14 | ((packed or bag* or sack* or home) adj5 (lunch* or food*)).mp. | 1733 |
| 15 | (lunch pail* or lunchpail*).mp. | 1 |
| 16 | ((box* adj lunch*) or boxlunch*).mp. | 19 |
| 17 | lunch*.mp. or Lunch/ | 4694 |
| 18 | 13 or 14 or 15 or 16 or 17 | 6231 |
| 19 | Randomized Controlled Trial/ | 406624 |
| 20 | Controlled Clinical Trial/ | 90068 |
| 21 | clinical trials as topic/ | 174956 |
| 22 | random*.tw. | 716136 |
| 23 | trial.tw. | 371430 |
| 24 | double-blind method/ or single-blind method/ | 153620 |
| 25 | (double blind or single blind).tw. | 118000 |
| 26 | experiment*.mp. | 1621342 |
| 27 | (pretest or pre test).mp. | 11278 |
| 28 | (posttest or post test).mp. | 11575 |
| 29 | (prepost or pre post).mp. | 4901 |
| 30 | before after.mp. | 3040 |
| 31 | qua?i randomi?ed.mp. | 2877 |
| 32 | stepped wedge.mp. | 153 |
| 33 | preference trial.mp. | 49 |
| 34 | comprehensive cohort*.mp. | 56 |
| 35 | natural experiment*.mp. | 1134 |
| 36 | qua?i experiment*.mp. | 6077 |
| 37 | randomi?ed encouragement trial*.mp. | 3 |
| 38 | (staggered enrolment trial* or staggered enrollment trial*).mp. | 0 |
| 39 | (non randomi?ed or nonrandomi?ed).mp. | 15346 |
| 40 | interrupted time series.mp. | 1186 |
| 41 | (time series and trial).mp. | 884 |
| 42 | multiple baseline.mp. | 1380 |
| 43 | regression discontinuity.mp. | 79 |
| 44 | 19 or 20 or 21 or 22 or 23 or 24 or 25 or 26 or 27 or 28 or 29 or 30 or 31 or 32 or 33 or 34 or 35 or 36 or 37 or 38 or 39 or 40 or 41 or 42 or 43 | 2727974 |
| 45 | 12 and 18 and 44 | 356 |
| **46** | **limit 45 to (english language and yr="1995 -Current")** | **302** |
